# Angiotensin II type I receptor (AT1R) is an independent prognosticator of esophageal squamous cell carcinoma and promotes cells proliferation via mTOR activation

**DOI:** 10.18632/oncotarget.11567

**Published:** 2016-08-24

**Authors:** Shau-Hsuan Li, Hung-I Lu, Alice Y.W. Chang, Wan-Ting Huang, Wei-Che Lin, Ching-Chang Lee, Wan-Yu Tien, Ya-Chun Lan, Hsin-Ting Tsai, Chang-Han Chen

**Affiliations:** ^1^ Department of Hematology-Oncology, Kaohsiung Chang Gung Memorial Hospital and Chang Gung University College of Medicine, Kaohsiung, Taiwan; ^2^ Department of Thoracic & Cardiovascular Surgery, Kaohsiung Chang Gung Memorial Hospital and Chang Gung University College of Medicine, Kaohsiung, Taiwan; ^3^ Institute of Physiology, National Cheng Kung University, Tainan, Taiwan; ^4^ Department of Pathology, Kaohsiung Chang Gung Memorial Hospital and Chang Gung University College of Medicine, Kaohsiung, Taiwan; ^5^ Department of Diagnostic Radiology, Kaohsiung Chang Gung Memorial Hospital and Chang Gung University College of Medicine, Kaohsiung, Taiwan; ^6^ Department of Gastroenterology, Kaohsiung Armed Forces General Hospital, Kaohsiung, Taiwan; ^7^ Institute of Basic Medical Sciences, National Cheng Kung University, Tainan, Taiwan, Taiwan; ^8^ Institute for Translational Research in Biomedicine, Kaohsiung Chang Gung Memorial Hospital, Kaohsiung, Taiwan; ^9^ Department of Applied Chemistry, and Graduate Institute of Biomedicine and Biomedical Technology, National Chi Nan University, Taiwan; ^10^ Center for Infectious Disease and Cancer Research, Kaohsiung Medical University, Kaohsiung, Taiwan

**Keywords:** esophageal cancer, squamous cell carcinoma, AT1R, angiotensin II, mTOR

## Abstract

**Background:**

The aim of this study was to investigate the effects of the angiotensin II/ angiotensin II type I receptor (AT1R) and angiotensin II type II receptor (AT2R) signaling pathway in esophageal squamous cell carcinoma (ESCC).

**Methods:**

Immunohistochemistry was performed to evaluate the expression levels of AT1R and AT2R in tissues from 152 surgically resected ESCC patients, and those expression levels were then correlated with treatment outcomes. The angiotensin II/AT1R/AT2R signaling pathway and its biological effects in the context of ESCC were investigated *in vitro* and *in vivo*.

**Results:**

In human samples, AT1R overexpression was univariately associated with inferior overall survival and remained multivariately independent (hazard ratio=1.812). *In vitro*, angiotensin II stimulated the growth of ESCC cells in a dose-dependent manner. Treatment with irbesartan or AT1R-RNAi knockdown but not treatment with PD123319 significantly decreased the level of angiotensin II-induced ESCC cell proliferation. Angiotensin II also caused mTOR activation in a dose-dependent manner, and everolimus or mTOR-RNAi knockdown significantly suppressed the level of angiotensin II-induced ESCC cell proliferation. Furthermore, AT1R-RNAi knockdown suppressed the activation of mTOR. Clinically, AT1R expression was also correlated with phosphorylated mTOR expression. In a xenograft model, local angiotensin II injection enhanced tumor growth, and this effect could be decreased by treatment with irbesartan or everolimus. In a 4-NQO-induced-ESCC murine model, irbesartan significantly decreased the incidence of esophageal tumor.

**Conclusions:**

These findings suggest that AT1R overexpression is an independent adverse prognosticator for patients with ESCC and that angiotensin II/AT1R signaling stimulates ESCC growth, in part through mTOR activation.

## INTRODUCTION

Esophageal cancer is currently the ninth leading cause of cancer death in Taiwan [1], and more than 90% of all cases of esophageal cancer consist of esophageal squamous cell carcinoma (ESCC) [2]. Although significant improvements have been made in treating this cancer via surgery and chemoradiotherapy, the prognosis for patients with ESCC remains poor [2, 3]. Therefore, the identification of new targets involved in the progression of ESCC is worthwhile, and for this, the elucidation of the underlying signaling pathway is critical.

Angiotensin II, a multifunctional bioactive octapep-tide and main effector of the renin-angiotensin system (RAS), plays a fundamental role as a vasoconstrictor in the control of cardiovascular function and renal homeostasis [4]. The majority of angiotensin II effects are mediated by the angiotensin II type 1 receptor (AT1R) and angiotensin II type 2 receptor (AT2R) [4]. Previously, it was thought that the RAS exists only in the circulatory system (circulating RAS). However, recent evidence suggests that the RAS exists at local tissue sites (local RAS), and such RAS has been implicated in various malignancies. AT1R and AT2R have been detected in several types of cancers [5, 6], and some have been reported to be associated with patients' prognosis [5, 6]. In addition, epidemiological studies may provide further evidence that angiotensin II signaling pathways influence tumor development and progression. Relatedly, Lever *et al*. reported the first clinical evidence that a long-term angiotensin II blockade might have a protective effect against carcinogenesis [7].

Although several experimental and epidemiological studies have suggested that angiotensin II/AT1R/AT2R may be involved in the development or progression of a variety of cancers, little is known about the role of the angiotensin II/AT1R/AT2R signaling pathway in ESCC. The aim of this study was thus to investigate the effects of the angiotensin II/AT1R/AT2R signaling pathway in ESCC.

## RESULTS

### Immunohistochemical expression of AT1R and AT2R and its correlations with other clinicopathologic parameters in ESCC

The clinicopathologic parameters of the 152 patients with ESCC are described in Table 1. The median age for the 152 patients, 146 men and 6 women, was 55 years (range, 29-80 years). The 7^th^ AJCC stages of 152 patients with ESCC were stage I in 43 patients, stage II in 66 patients, stage III in 39 patients, and stage IV in 4 patients. The tumor locations were upper in 19 patients, middle in 60 patients, and lower in 73 patients. The histological grades were grade 1 in 18 patients, grade 2 in 91 patients, and grade 3 in 43 patients. At the time of analysis, the median periods of follow-up were 64 months (range, 34-146 months) for the 56 survivors and 34 months (range, 3.5-146 months) for all 152 patients. The 5-year overall and disease-free survival rates of these 152 patients were 43% and 39%, respectively.

**Table 1:** Clinicopathologic features of 152 patients with esophageal squamous cell carcinoma

Parameters	No. of cases (percentage)
Median age (range)	55 (29–80)
Sex	
Male	146 (96%)
Female	6 (4%)
T classification	
T1	46 (30%)
T2	32 (21%)
T3	59 (39%)
T4	15 (10%)
N classification	
N0	102 (67%)
N1	31 (20%)
N2	13 (9%)
N3	6 (4%)
7^th^ AJCC staging	
I	43 (28%)
II	66 (44%)
III	39 (26%)
IV	4 (2%)
Histological grading	
1	18 (12%)
2	91 (60%)
3	43 (28%)
Location	
Upper	19 (13%)
Middle	61 (40%)
Lower	72 (47%)
Keratinizing	
Absence	26 (17%)
Presence	126 (83%)
Surgical margin	
Negative	133 (88%)
Positive	19 (12%)
AT1R expression	
Low expression	92 (61%)
Overexpression	60 (39%)
AT2R expression	
Low expression	89 (59%)
Overexpression	63 (41%)
p-mTOR expression	
Low expression	71 (47%)
Overexpression	81 (53%)
t-mTOR expression	
Low expression	53 (35%)
Overexpression	99 (65%)

AJCC, American Joint Committee on Cancer; AT1R, angiotensin II type 1 receptor; AT2R, angiotensin II type 2 receptor; p-mTOR, phosphorylated mammalian target of rapamycin; t-mTOR, total mammalian target of rapamycin.

**Figure 1:**
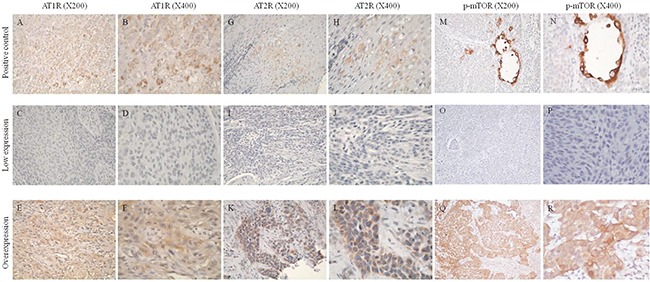
Immunohistochemical staining of angiotensin II type I receptor (AT1R), angiotensin II type II receptor (AT2R), and phosphorylated mammalian target of rapamycin (p-mTOR) **A, B.** AT1R immunoreactivity was present in normal adrenal gland tissues used as a positive control. **C, D.** Low expression of AT1R. **E, F.** Overexpression of AT1R. **G, H.** AT2R immunoreactivity was present in normal adrenal gland tissues used as a positive control. **I, J.** Low expression of AT2R. **K, L.** Overexpression of AT2R. **M, N.** p-mTOR immunoreactivity was present in normal submucosal glands from the esophagus used as a positive control. **O, P.** Low expression of p-mTOR. **Q, R.** Overexpression of p-mTOR.

The correlations between the clinicopathological parameters and immunohistochemical expressions of AT1R and AT2R (Figure 1) are summarized in Table 2. AT1R overexpression was significantly associated with high pathologic T stage (T3+4; P<0.001) and advanced 7^th^ AJCC staging (stage II+III+IV; P=0.028). AT2R overexpression was significantly associated with high pathologic T classification (T3+4; P=0.016) and AT1R overexpression (P=0.001).

**Table 2:** Associations between AT1R, AT2R, p-mTOR expression, t-mTOR expression, and clinicopathologic factors in 152 patients with esophageal squamous cell carcinoma receiving esophagectomy

Parameters		AT1R expression			AT2R expression			p-mTOR expression			t-mTOR expression		
		Low	Over	P value	Low	Over	P value	Low	Over	P value	Low	Over	P value
Age													
	< 55y/o	40	29	0.56	42	27	0.60	33	36	0.80	24	45	0.98
	≥55y/o	52	31		47	36		38	45		29	54	
Sex													
	Male	88	58	1.00	85	61	1.00	66	80	0.098	52	94	0.67
	Female	4	2		4	2		5	1		1	5	
T classification													
	T1+2	59	19	<0.001*	53	25	0.016*	41	37	0.14	24	54	0.28
	T3+4	33	41		36	38		30	44		29	45	
N classification													
	N0	63	39	0.66	62	40	0.43	53	49	0.064	36	66	0.88
	N1+2+3	29	21		27	23		18	32		17	33	
7^th^ AJCC staging													
	I	32	11	0.028*	30	13	0.078	24	19	0.16	11	32	0.13
	II+III+IV	60	49		59	50		47	62		42	67	
Histological grading													
	1+2	71	38	0.064	63	46	0.76	52	57	0.70	40	69	0.45
	3	21	22		26	17		19	24		13	30	
Location													
	U+M	49	31	0.85	50	30	0.30	41	39	0.24	28	52	0.97
	L	43	29		39	33		30	42		25	47	
Keratinizing													
	Absent	15	11	0.75	18	8	0.23	12	14	0.95	9	17	0.98
	Present	77	49		71	55		59	67		44	82	
Surgical margin													
	Negative	81	52	0.80	75	58	0.15	65	68	0.16	44	89	0.22
	Positive	11	8		14	5		6	13		9	10	
AT1R expression													
	Low	-	-	-	64	28	0.001*	54	38	<0.001*	28	64	0.16
	Over	-	-		25	35		17	43		25	35	
AT2R expression													
	Low	64	25	0.001*	-	-	-	-	-	-	30	59	0.72
	Over	28	35		-	-		-	-		23	40	
p-mTOR expression													
	Low	54	17	<0.001*	46	25	0.14	-	-	-	26	45	0.67
	Over	38	43		43	38		-	-		27	54	
t-mTOR expression													
	Low	28	25	0.16	30	23	0.72	26	27	0.67	-	-	-
	Over	64	35		59	40		45	54		-	-	

AJCC, American Joint Committee on Cancer; AT1R, angiotensin II type 1 receptor; AT2R, angiotensin II type 2 receptor; p-mTOR, phosphorylated mammalian target of rapamycin; t-mTOR, total mammalian target of rapamycin;

* Statistically significant; x2 test, Fisher's exact test, or t test was used for statistical analysis.

### AT1R overexpression is an independent adverse prognosticator for patients with ESCC

**Figure 2:**
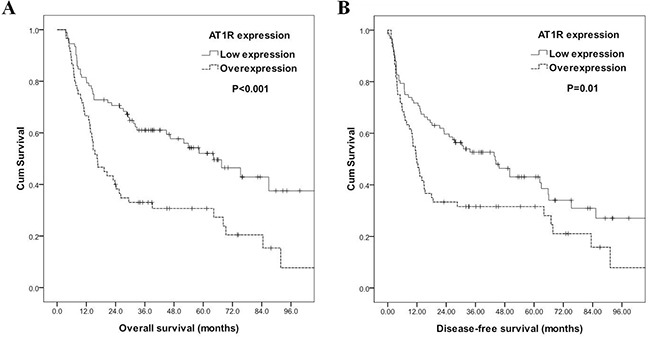
Kaplan–Meier curves according to angiotensin II type I receptor (AT1R) status. **A.** Overall survival according to AT1R status **B.** Disease-free survival according to AT1R status.

Correlations of the clinicopathological parameters and the AT1R and AT2R expression levels with overall survival and disease-free survival are shown in Table 3. Univariate analyses demonstrated that 7^th^ AJCC stage II+III+IV (P<0.001), T3+4 disease (P<0.001), positive regional lymph node (P<0.001), grade 3 (P=0.011), positive surgical margin (P=0.017), and AT1R overexpression (P<0.001, Figure 2A) were significantly associated with inferior overall survival. Additionally, 7^th^ AJCC stage II+III+IV (P<0.001), T3+4 disease (P<0.001), positive regional lymph node (P<0.001), grade 3 (P=0.04), positive surgical margin (P=0.035), and AT1R overexpression (P=0.01, Figure 2B) were significantly associated with inferior disease-free survival. In a multivariate comparison, AT1R overexpression (P=0.004, hazard ratio=1.812, 95% confidence interval: 1.207-2.720) remained independently associated with worse overall survival, together with 7^th^ AJCC stage II+III+IV (P<0.001, hazard ratio=3.120, 95% confidence interval: 1.788-5.445). For disease-free survival, AT1R overexpression (P=0.025, hazard ratio=1.567, 95% confidence interval: 1.058-2.323) and 7^th^ AJCC stage II+III+IV (P<0.001, hazard ratio=2.623, 95% confidence interval: 1.585-4.339) represented an independent adverse prognosticator.

**Table 3:** Results of univariate log-rank analysis of prognostic factors for overall survival and disease-free survival in 152 patients with esophageal squamous cell carcinoma receiving esophagectomy

Parameters	No. of patients	Overall survival (OS)		Disease-free survival (DFS)	
		5-yr OS rate (%)	P value	5-yr DFS rate (%)	P value
Age					
< 55y/o	69	50%	0.30	49%	0.12
≥55y/o	83	36%		32%	
T classification					
T1+2	78	59%	<0.001*	52%	<0.001*
T3+4	74	25%		26%	
N classification					
N0	102	55%	<0.001*	51%	<0.001*
N1+2+3	50	17%		16%	
7^th^ AJCC staging					
I	43	73%	<0.001*	65%	<0.001*
II+III+IV	109	31%		29%	
Histological grading					
Grade 1+2	109	49%	0.011*	44%	0.04*
Grade 3	43	27%		27%	
Location					
Upper/middle	80	44%	0.90	39%	0.69
Lower	72	41%		40%	
Keratinizing					
Absent	26	54%	0.36	50%	0.28
Present	126	40%		37%	
Surgical margin					
Negative	133	45%	0.017*	41%	0.035*
Positive	19	25%		25%	
AT1R expression					
Low expression	92	51%	<0.001*	46%	0.01*
Overexpression	60	30%		30%	
AT2R expression					
Low expression	89	46%	0.18	40%	0.51
Overexpression	63	38%		38%	
p-mTOR expression					
Low expression	71	57%	0.012*	53%	0.018*
Overexpression	81	29%		27%	
t-mTOR expression					
Low expression	53	50%	0.70	47%	0.83
Overexpression	99	38%		36%	

AJCC, American Joint Committee on Cancer; AT1R, angiotensin II type 1 receptor; AT2R, angiotensin II type 2 receptor; p-mTOR, phosphorylated mammalian target of rapamycin; t-mTOR, total mammalian target of rapamycin;

* Statistically significant

### Angiotensin II stimulates ESCC cell growth via AT1R

**Figure 3:**
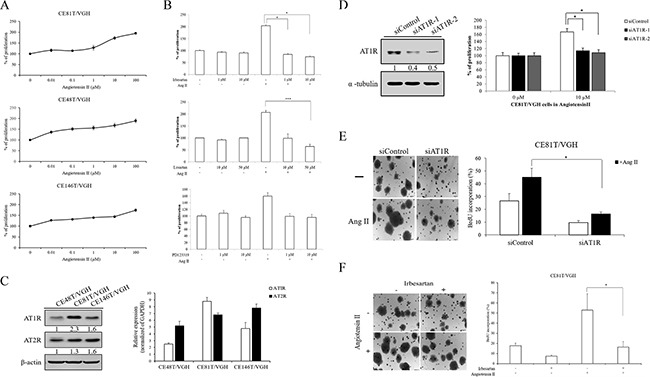
Angiotensin II induced cell proliferation of ESCC was required for AT1R **A.** Serum-starved CE81T/VGH, CE48T/VGH, and CE146T/VGH cells treated with or without angiotensin II stimulation were seeded into 96-well plates with 1.0% of FBS. The cells were cultured for 72 hours followed by MTT assay (OD570) to quantitate cell growth. The data were normalized against the OD570 value on control group (0 μM) of each treatment. **B.** Serum-starved cells were pre-treated with or without various concentrations of irbesartan or losartan or PD123319 for 30 mins; the cells were then stimulated with angiotensin II (10 μM). The cells were cultured for 72 hours followed by MTT assay to quantitate cell growth. **C.** The mRNA and protein expression profiles of AT1R and AT2R in ESCC cell lines were determined by Q-RT-PCR and Western blotting. **D** and **E.** The abilities of cell growth, colony formation, and BrdU incorporation in AT1R-depleted CE81T/VGH cells or siControl group with or without angiotensin II (10 μM) stimulation were assayed. **F.** The abilities of colony formation and BrdU incorporation in angiotensin II-stimulated CE81T/VGH cells treated with or without irbesartan were assayed.

In order to determine the dose–response effect of angiotensin II on cell growth, CE81T/VGH, CE48T/VGH, and CE146T/VGH cells were treated with angiotensin II (10^−8^ to 10^−4^M) for 72 h. Angiotensin II stimulated the growth of all three types of ESCC cells in a dose-dependent manner (Figure 3A). In order to determine if AT1R or AT2R is involved in angiotensin-II-induced ESCC cell proliferation, CE81T/VGH cells were treated with an AT1R antagonist (irbesartan or losartan) or an AT2R antagonist (PD123319) for 30 min prior to the angiotensin II treatment. The AT1R antagonists significantly decreased the level of angiotensin II-induced ESCC cell proliferation (P<0.05, Figure 3B), whereas the AT2R antagonist had no effect (P>0.05, Figure 3B). Similar results were also observed in CE48T/VGH (Supplementary Figure S1A). To determine if AT1R and AT2R were expressed in ESCC cell lines, the mRNA and protein expression levels of both AT1R and AT2R were determined by Q-RT-PCR and Western blotting in three ESCC cell lines. The data indicated that both AT1R and AT2R were expressed in the ESCC cells (Figure 3C). Moreover, the mRNA levels of both AT1R and AT2R were consistent with the protein profiles. To further confirm the role of AT1R in angiotensin II-induced ESCC cell proliferation, siRNA was used to reduce the endogenous AT1R expression in ESCC cells. The endogenous protein expression levels of AT1R in CE81T/VGH and CE48T/VGH cells transfected with siRNAs targeting AT1R were significantly reduced (Figure 3D and Supplementary Figure S1B). The knockdown of endogenous AT1R in CE81T/VGH or CE48T/VGH cells led to a significant decrease of angiotensin II-induced ESCC cell proliferation, colony formation, and BrdU incorporation (P<0.05, Figure 3D and 3E and Supplementary Figure S1B and S1C). Additionally, we also demonstrated that CE81T/VGH treated with irbesartan caused a dramatic reduction of angiotensin II-induced colony formation and BrdU incorporation (Figure 3F). Collectively, these results suggest that angiotensin II stimulates ESCC cell growth through AT1R.

### Angiotensin II/AT1R signaling stimulates mTOR pathway

**Figure 4:**
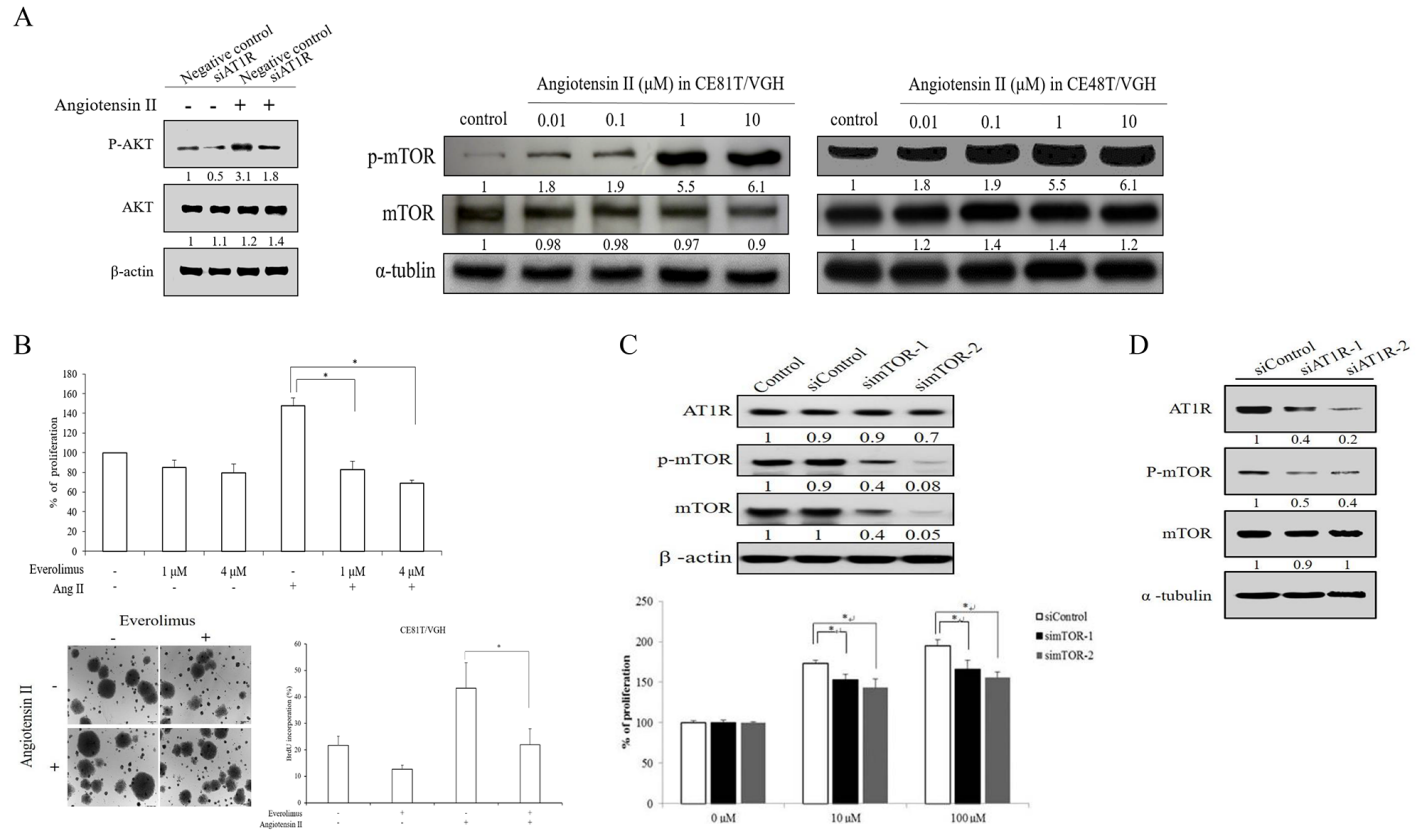
mTOR expression and activation participated in angiotensin II/AT1R signaling in ESCC (**A**, left panel) The AKT activation was examined in AT1R-depleted cells with or without angiotensin II stimulation. (**A**, right panel) the phosphorylated status of mTOR was determined in ESCC cells stimulated with angiotensin II by Western blotting. **B.** Serum-starved cells were pre-treated with indicated concentrations of everolimus for 30 mins; the cells were then stimulated with or without angiotensin II. The cells were cultured for 72 hours followed by MTT assay to quantitate cell growth. In addition, the abilities of colony formation and BrdU incorporation in angiotensin II-stimulated CE81T/VGH cells treated with or without everolimus were assayed. **C.** The protein expression levels of total mTOR, phosphorylated mTOR and AT1R were demonstrated in CE81T/VGH cells transfected with siControl and simTOR. The cell growth abilities of siControl and simTOR stimulated with angiotensin II were measured by MTT assay. **D.** The protein expression profiles of AT1R, total mTOR, and phosphorylated mTOR were determined in AT1R-depleted CE81T/VGH cells.

To further study the mechanisms underlying the growth promoting effects of angiotensin II, we investigated the downstream molecules of angiotensin II/AT1R signaling. We first examined if MAPK expression was altered in AT1R-depleted ESCC cells in the absence or presence of angiotensin II. Western blotting showed that phosphorylated AKT was dramatically reduced in AT1R-depleted cells compared to negative control. Moreover, AKT activation was slightly increased in siAT1R cells treated with angiotensin II (Figure 4A). However, the activations of ERK, JNK, and p38 were not influenced in loss-of-function-of-AT1R ESCC cells with or without angiotensin II stimulation (data not shown). Additionally, Western blotting analyses also showed that angiotensin II stimulated p-mTOR expression in CE81T/VGH and CE48T/VGH cells in a dose-dependent manner (Figure 4A). CE81T/VGH and CE48T/VGH cells were treated with everolimus (an mTOR inhibitor) prior to the angiotensin II treatment, and everolimus significantly decreased the level of angiotensin II-induced ESCC cell proliferation, colony formation, and BrdU incorporation (Figure 4B). The proliferative inhibition of CE48T/VGH cells treated with everolimus upon angiotensin II stimulation was also observed (Supplementary Figure S2A). To further confirm the role of mTOR activation in angiotensin II-induced ESCC cell proliferation, siRNA was used to reduce endogenous mTOR expression in CE81T/VGH and CE48T/VGH cells. The endogenous protein expression levels of mTOR in CE81T/VGH or CE48T/VGH cells transfected with siRNAs targeting mTOR were significantly reduced (Figure 4C and Supplementary Figure S2B). The knockdown of endogenous mTOR in CE81T/VGH and CE48T/VGH cells led to a significant decrease in the angiotensin II-induced ESCC cell proliferative effect (P<0.05; Figure 4C and Supplementary Figure S2B). Additionally, the knockdown of endogenous AT1R in CE81T/VGH and CE48T/VGH cells also led to a decrease in the p-mTOR levels measured by Western blotting (Figure 4D and Supplementary Figure S2C). These findings suggest that angiotensin II/AT1R signaling stimulates ESCC cell growth through mTOR activation. To evaluate the potential relevance of the above findings in ESCC in clinical practice, we also analyzed the protein expressions of p-mTOR and t-mTOR in tissues from 152 human ESCC patients using immunohistochemistry, and there was a significant correlation between AT1R expression and p-mTOR expression (P<0.001, Table 2). Representative staining results of low expression and overexpression are shown in Figure 1.

### Angiotensin II injection enhanced ESCC xenograft growth, and this effect could be decreased by irbesartan or everolimus

**Figure 5:**
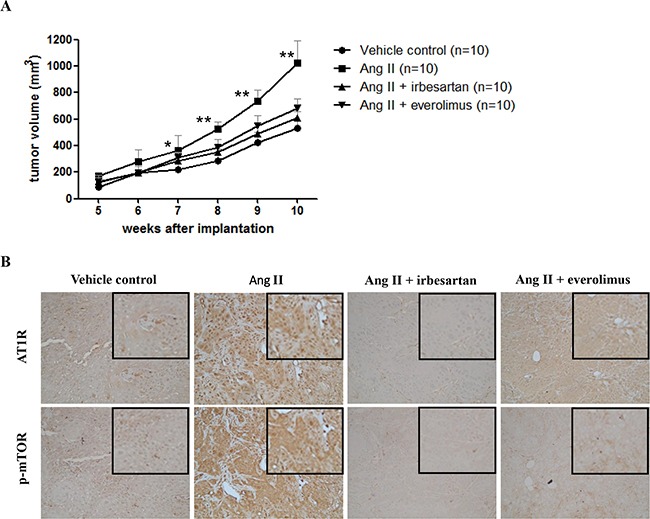
Angiotensin II injection enhanced ESCC xenograft growth, and this effect could be decreased by irbesartan or everolimus **A.** The graphs show tumor volumes with time after treatment with vehicle control, angiotensin II, angiotensin II plus irbesartan, and angiotensin II plus everolimus, respectively. *Significant difference between angiotensin II group and vehicle control group (P<0.05). **Significant difference between angiotensin II group and angiotensin II plus irbesartan, angiotensin II plus everolimus, and vehicle control groups (P<0.05). **B.** The immunohistochemistry of AT1R and p-mTOR in xenograft tumors in vehicle control, angiotensin II, angiotensin II plus irbesartan, and angiotensin II plus everolimus groups. The magnified figures are shown in the upper right corner. Original magnification ×200.

Our *in vitro* data showed that angiotensin II can stimulate ESCC cell growth. Therefore, we tested whether the proliferative effect of angiotensin II exists in an ESCC xenograft model. As shown in Figure 5A, angiotensin II significantly promoted CE81T/VGH xenograft tumor growth compared to a vehicle control group. In addition, adding irbesartan or everolimus significantly suppressed the promotion effect of angiotensin II in CE81T/VGH xenografts (Figure 5A). Compared to the angiotensin II group, the immunohistochemistry of xenograft tumors revealed lower AT1R expression in the vehicle control and irbesartan groups, as well as lower p-mTOR expression in the vehicle control, irbesartan, and everolimus groups (Figure 5B).

### Irbesartan significantly decreased the incidence of esophageal tumor in 4-NQO-induced ESCC murine model

**Figure 6:**
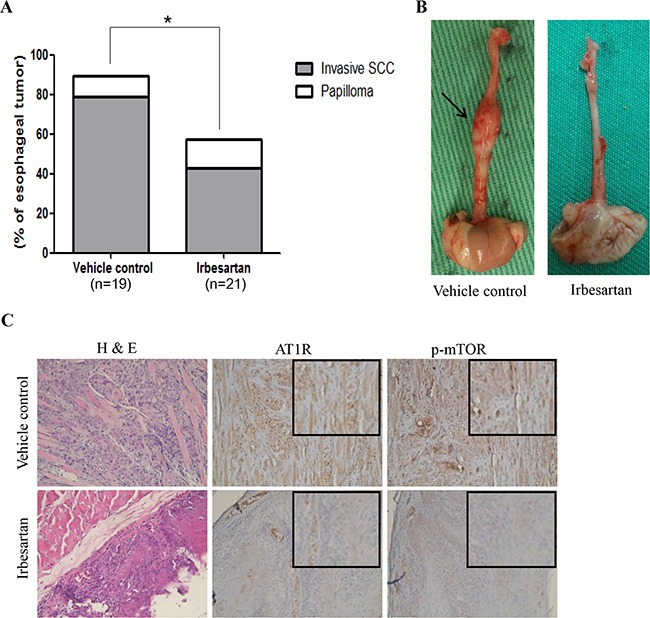
Inhibitory effect of irbesartan in 4-NQO-induced ESCC murine model **A.** The incidence of esophageal tumor in mice treated with irbesartan was significantly lower than that in mice treated with vehicle control (57% versus 89%; P = 0.034). **B.** Gross appearance of esophagus from representative mice treated with irbesartan or vehicle control. The arrows indicate an enlargement of esophageal tumor. **C.** Hematoxylin and eosin stained (H&E) sections from representative mice treated with vehicle control showed esophageal squamous cell carcinoma with muscle invasion. H&E sections from representative mice treated with irbesartan showed only esophageal dysplasia. Compared to the vehicle control group, immunohistochemistry revealed lower AT1R and p-mTOR expression in the irbesartan group. The magnified figures are shown in the upper right corner. Original magnification ×200. SCC: squamous cell carcinoma.

We next determined whether the inhibitory effect of the AT1R antagonist, irbesartan, exists in a 4-NQO-induced ESCC murine model. Among the mice treated with vehicle control, 17 (89%) of the 19 mice developed esophageal tumors, including invasive squamous cell carcinoma in 15 mice and papilloma in 2 mice. Among the mice treated with irbesartan, 12 (57%) of the 21 mice developed esophageal tumors, including invasive squamous cell carcinoma in 9 mice and papilloma in 3 mice. The incidence of esophageal tumor in mice treated with irbesartan was significantly lower than that in mice treated with vehicle control (57% versus 89%; P = 0.034; Figure 6A & 6B). Compared to the vehicle control group, immunohistochemistry revealed low AT1R and p-mTOR expression in the irbesartan group (Figure 6C).

## DISCUSSION

Given the expression of the RAS in many tissues, it is perhaps not surprising that many components of the RAS are also expressed in malignant tissue. Indeed, many studies have reported that AT1R overexpression in tumor tissues is common in a number of cancers (such as prostate, lung, kidney, pancreas, and breast cancers, among others) [5], and may be correlated with tumor progression and the prognoses of such patients [5, 8]. But, to the best of our knowledge, the significance of AT1R expression in ESCC remains unclear. Our study results showed that AT1R protein overexpression was associated with higher T classification, advanced 7^th^ AJCC staging, and worse overall survival. In specific cell lines, treatment with an AT1R antagonist or AT1R siRNA can block angiotensin-II-induced ESCC cell proliferation. These results suggest an involvement of AT1R in ESCC progression and a potential therapeutic effect of receptor antagonists in the treatment of ESCC.

Angiotensin II is a main effector peptide in the RAS and can stimulate tumor growth via the AT1R [9]. A growing body of evidence suggests that the stimulation of angiotensin II-mediated AT1R activation gives rise to the encouragement of various intracellular cascades of protein kinases, such as MAP/ERK kinase, PI3K, protein kinase C, and JAK-STAT3 [10, 11]. Furthermore, angiotensin II-AT1R signaling could also increase cell proliferation through cooperation with EGFR signaling [12]. In contrast, several previous studies demonstrated that AT1R expression was low and caused decreased or no significant regulation of cell growth in response to angiotensin II stimulation in some cancer cells [13–15]. Taken together, these results indicated that the role of angiotensin II-AT1R signaling in cell growth remains controversial and contradictory in a variety of human cancer cells. In the present study, we illustrated that the growth capability of ESCC was increased under angiotensin II stimulation in a dose-dependent manner both *in vitro* and *in vivo*. In addition, the suppression of endogenous AT1R by AT1R siRNAs following treatment with angiotensin II inhibited cell growth, colongenic formation, and BrdU incorporation of ESCC, compared to siControl growth with angiotensin II stimulation. Taken together, these results indicate that angiotensin II and AT1R stimulate tumor growth in ESCC.

It is well-known that the angiotensin II signaling pathway is involved in the vascular and renal systems. Clinically, inhibitors of the angiotensin II signaling pathway, such as AT1R blockers, are widely used in the treatment of hypertension, especially for patients with diabetes, heart failure, or stroke, and have few critical side effects [16, 17]. This large population of patients further supports the importance of elucidating the role of the angiotensin II signaling pathway in various malignancies. The relationship between the use of AT1R blockers and cancer risk has drawn significant attention since 2010 [18]. Accumulating evidence has shown that AT1R blockers are associated with decreased risks of colorectal cancer, lung cancer, and prostate cancer [19, 20]. However, some studies have also found that there is no significant association between the use of AT1R blockers and other human cancers, such as liver, gastric, and hematological malignancies [16, 21]. These inconsistent results have indicated that advanced studies and data collection involving different types of cancers will be required in order to identify the exact effects of AT1R blockers. Accumulating evidence has further indicated that MAPK proteins, such ERK, JNK, p38, and AKT, are involved in AT1R signaling in human cancer cells. [22] In the present results, we found that only AKT activation was significantly reduced in AT1R-depleted ESCC cell lines, suggesting that AKT plays an essential role in AT1R signaling that elicits cancer cell progression in ESCC. It is also known that mTOR, a key downstream target of the PI3K/AKT pathway, is an important controller of cell growth. Thus, mTOR can be taken as a central marker for targeted therapies aimed at human cancer cells. [23, 24] In clinical practice, the mTOR inhibitors have also been approved for the treatment of advanced renal cell carcinoma, pancreatic neuroendocrine tumors, and hormone receptor-positive breast cancer [25]. The finding of a new therapeutic use for a proven drug has the advantage of decreased development costs and decreased time to market compared to traditional discovery efforts because of the availability of previously collected pharmacokinetic, toxicology, and safety data. In the present study, we found that angiotensin II-elicited ESCC cell growth was restrained in cells treated with AT1R blockers both in vitro and in vivo. We also found that mTOR expression was increased in ESCC cells under angiotensin II stimulation in a dose-dependent manner, and that the angiotensin II-enhanced growth capability of ESCC cells was reduced in cells treated with mTOR inhibitors. Our results suggest that angiotensin II/AT1R/mTOR signaling may be a potential therapeutic target for treating ESCC.

Our study has important limitations. First, the present study was a retrospective analysis. Second, our observations were limited by the relatively small number of patients. In conclusion, AT1R overexpression is independently associated with poor prognosis in patients with ESCC. In addition, angiotensin II/AT1R signaling stimulates ESCC growth, in part through mTOR activation. Therefore, angiotensin II/AT1R signaling could provide a novel therapeutic target for the treatment of ESCC.

## MATERIALS AND METHODS

### Reagents

Human angiotensin II, irbesartan, losartan, PD123319, and everolimus were purchased from Sigma (Sigma Aldrich, St Louis, MO, USA).

### Patient population

Patients with ESCC who underwent surgical resection at Kaohsiung Chang Gung Memorial Hospital were retrospectively reviewed. This study was approved by the institutional review board of Chang Gung Memorial Hospital. Patients with synchronous cancers in other organs and patients receiving preoperative chemoradiotherapy, preoperative chemotherapy, or preoperative radiotherapy were excluded. Finally, 152 patients with available paraffin blocks and follow-up were identified. Patients undergoing surgery had a radical esophagectomy with cervical esophagogastric anastomosis (McKeown procedure) or an Ivor Lewis esophagectomy with intrathoracic anastomosis, reconstruction of the digestive tract with gastric tube, and pylorus drainage procedures. Two-field lymph node dissection was performed in all patients. The pathological TNM stage was determined according to the 7^th^ American Joint Committee on Cancer (AJCC) staging system [26]. Overall survival (OS) was calculated from the time of surgery to death as a result of all causes. Disease-free survival (DFS) was computed from the time of surgery to the recurrence or death from any cause without evidence of recurrence.

### Immunohistochemistry

Immunohistochemistry was done as previously described [27]. Antibodies used in immunohistochemistry are AT1R (sc-1173, 1:50, Santa Cruz Biotechnology, Santa Cruz, CA, USA), AT2R (sc-7421, 1:50, Santa Cruz Biotechnology, Santa Cruz, CA, USA), phosphorylated-mTOR (p-mTOR) (Ser2448, Clone 49F9, 1:50, Cell Signaling Technology, Boston, MA, USA), and total-mTOR (t-mTOR) (1:50, Cell Signaling Technology, Boston, MA, USA). For AT1R and AT2R, incubation without the primary antibody was used as a negative control, while normal adrenal gland [28–30] was used as a positive control. For p-mTOR and t-mTOR, incubation without the primary antibody and normal esophageal squamous epithelium [31] were used as a negative control, while normal submucosal gland from esophagus and normal gastric gland [31] were used as a positive control. The staining assessment was independently carried out by two pathologists (S.L.W. and W.T.H) without any information about clinicopathologic features or prognosis. We followed the previously published method to score the expression of AT1R, AT2R, p-mTOR, and t-mTOR [5, 31, 32]. The percentage of AT1R, AT2R, p-mTOR, or t-mTOR positive tumor cells of all neoplastic cells in the section was recorded. The AT1R or AT2R overexpression was defined as the presence of at least staining in ≧35% of tumor cells [5, 32]. The p-mTOR or t-mTOR overexpression was defined as the presence of at least staining in ≧10% of tumor cells [31].

### RNA extraction and real-time PCR assay

Total RNA isolated from cells was obtained by using Trizol reagent. Total RNA (2 μg) was reverse transcribed in a final volume of 20 μl using random primers under standard conditions for the PrimeScript RT reagent Kit. We used the Taq-Man probe to detect the expression levels of AT1R and AT2R, following the manufacturer's instructions. Results were normalized to GAPDH.

### Immunoblot analysis

For cell protein extraction, samples were homoge-nized in RIPA lysis buffer (50 mM Tris-HCl, pH 7.5, 150 mM NaCl, 1% NP-40, 0.5% Na-deoxycholate, and 0.1% SDS). The protein concentration in each sample was estimated by Bio-Rad Protein Assay (Bio-Rad, Hercules, CA, USA). Immunoblotting was performed according to standard procedures. Antibodies used in this study include polyclonal antibodies against AT1R Cruz Biotechnology, Santa Cruz, CA, USA), p-mTOR (Ser2448, Clone 49F9, Cell Signaling Technology, Boston, MA, USA), mTOR (Clone 7C10, Cell Signaling Technology, Boston, MA, USA), β-actin (Sigma Aldrich, St Louis, MO, USA), and α-tubulin (GENE TEX). The first antibodies were detected by incubation with secondary antibodies conjugated to HRP (Bio/Can Scientific, Mississauga, ON, Canada) and developed using Western Lighting Reagent. The proteins were explored by X-ray films.

### Cell culture and transient transfection

The CE81T/VGH and CE48T/VGH ESCC cell lines were obtained from the Bioresource Collection and Research Center (BCRC), and cultured in Dulbecco's Modified Eagle Medium (DMEM) supplemented with 10% fetal calf serum, 2 mmol/L glutamine, 100 U/mL penicillin, and 100 μg/mL streptomycin. The CE146T/VGH cell line was also obtained from the BCRC and cultured in Roswell Park Memorial Institute (RPMI)-1640 Medium supplemented with 10% fetal calf serum, 2 mmol/L glutamine, 100 U/mL penicillin, and 100 μg/mL streptomycin. The siRNAs specifically against the AT1R or mTOR were purchased from Dharmacon. siRNAs were transiently transfected into cancer cells using Lipofectamine (Invitrogen) according to the manufacturer's instructions. Next, the cells were then harvested and analyzed for endogenous AT1R, mTOR, or p-mTOR expressions by Western blotting. Four double-stranded synthetic RNA oligomers (5′-CAAAGGACUUCGCCCAUAAtt-3′ and 5′-GCAGAAUUGUCAAGGGAUAtt-3′) deduced from human *mTOR*, (5′-GAAGCUGAAGACUGUGGCCAGUGUtt-3′ and 5′-ATTGGGTGAACAATAGtt-3′) deduced from human *AT1R*, and one negative control siRNA were used in the siRNA experiments.

### Cell viability, colony formation assay and BrdU incorporation assays

The viability of sub-confluent cells was analyzed by 3-(4,5-dimethylthiazole-2-yl)-2,5-diphenyltetrazolium bromide (MTT) reduction assay. Cells were seeded at 5 × 10^3^ cells/well in 96-well plates. The next day, cells were treated with irbesartan, losartan, PD123319, and everolimus for 72 h. Then the culture medium was removed and the cells were washed with fresh culture medium without FBS. Cells were then incubated with 0.5 mg/ml MTT, in culture medium without FBS, for 4 h at 37°C in a 5% CO_2_ atmosphere. The medium was removed and 100 μL DMSO buffer was added and incubated in the dark for 10 min. Absorbance was measured on a microplate reader at 540 nm. The OD values were normalized with the value for the control group. For colony formation assay, negative control or siAT1R cells were seeded in 60-mm dishes at a density of 5 × 10^3^ cells. Next day, cells were treated with Angiotensin II for 72 h. After 21 days, cell colonies were counted after staining with 0.01 % crystal violet. For bromodeoxyuridine (BrdU) incorporation assay, cells were seeded at a density of 5 × 10^3^ cells/well in 96-well culture plates. Next day, cells were starved in DMEM for 24 h to achieve synchronous growth, and then exposed to the 10 μmol/L of BrdU. BrdU incorporation analysis was performed using the cell proliferation ELISA kit (Roche Diagnostics, Mannheim, Germany) according to the manufacturer's instructions.

### Nude mouse xenograft model

Six-week-old male athymic nude mice were used, and all animal experiments conformed to the protocols approved by the Experimental Animal Committee of Chang Gung Memorial Hospital. CE81T/VGH cells (2×10^6^ cells per implantation, five mice per group) were implanted subcutaneously on the dorsal gluteal region. Measurements of tumor volume with calipers were started one week after implantation and performed weekly. Tumor volume was calculated from the following formula: tumor volume (mm^3^) = length × width^2^/2, where length and width were the longer and shorter dimensions of the tumor, respectively. One week after implantation, the mice were divided into four groups. Each group had ten mice, and treated as follows. Group 1, local 10μl angiotensin II (10^−5^ M) injection at implantation site for 5 days/wk; group 2, local 10μl PBS injection at implantation site for 5 days/wk; group 3, local 10μl angiotensin II (10^−5^ M) injection at implantation site plus irbesartan 50 mg/kg/d [33] by oral gavage for 5 days/wk; and group 4, local 10μl angiotensin II (10^−5^ M) injection at implantation site for 5 days/wk plus everolimus 5mg/kg [34] by oral gavage twice a week.

### 4-nitroquinoline 1-oxide (4-NQO)-induced ESCC murine model

The carcinogen, 4-NQO (Sigma Aldrich, St Louis, MO, USA) stock, was first dissolved in DMSO at 50 mg/mL as a stock solution and stored at -20°C until used. On the days of 4-NQO administration, the stock solution was dissolved in propylene glycol (Sigma Aldrich, St Louis, MO, USA) and added to the drinking water bottles containing autoclaved tap water to obtain a final concentration of 100 μg/mL. Fifty six-week-old C57Bl/6 mice were treated with 100μg/ml 4-NQO in drinking water for 16 weeks and then resumed with normal autoclaved drinking water for another 12 weeks (total 28 weeks). The treatment of irbesartan (50 mg/kg/d for 5 days/wk for 10 weeks by oral gavage)[33] or vehicle control was started 16 weeks after initiation of 4-NOQ treatment, and each group had 25 mice. Mice dying prior to the end of the experiment were excluded from the analysis. At the end of the experiment, mice were subjected to autopsies; whole esophagus and stomach will be opened longitudinally, and macroscopic lesions were observed and identified carefully. For histological examination, the esophagus and gross lesions were fixed in 10% buffered formalin, embedded in paraffin blocks and then the histological sections were stained with hematoxylin and eosin. The histological determination were performed by two pathologists (S.L.W. and W.T.H) according to the criteria described previously [35, 36]. Hyperplasia was defined as thickened epithelium with prominent surface keratinization and with or without elongated rete ridges. Dysplasia was defined as loss of polarity in the epithelial cells, nuclear pleomorphism and hyperchromasia, abnormal single cell keratinization (dyskeratosis), and increased or abnormal mitoses. Esophageal tumors were histopathologically papilloma or invasive squamous cell carcinoma. Papilloma was defined as noninvasive exophytic growth of neoplastic cells, and invasive squamous cell carcinoma was defined as a lesion with invasion into the subepithelial tissues.

### Statistical analysis

For patient data, statistical analysis was performed using the SPSS 17 software package. The Chi-square test and Fisher's exact test were employed to compare data between the two groups. For survival analysis, the Kaplan–Meier method was used for univariate analysis, and the difference between survival curves was tested by a log-rank test. In a stepwise forward fashion, significant parameters at univariate level were entered into Cox regression model to analyze their relative prognostic importance. For cell line experiments, *t* test was used for the statistical analysis. Each experiment was carried out independently at least twice, with three repeats each. For nude mouse xenograft experiments, tumor volumes were compared using the two-way analysis of variance followed by Bonferroni's post-hoc test. For 4-NQO-induced ESCC mice experiments, statistical analyses of the incidence of esophageal tumors were performed using Fisher's exact test. For all analyses, a P value < 0.05 was considered statistically significant.

## SUPPLEMENTARY MATERIAL FIGURES


