# Supplementary material for: Angiotensin II type I receptor (AT1R) is an independent prognosticator of esophageal squamous cell carcinoma and promotes cells proliferation via mTOR activation
Source: Oncotarget. 2016 Aug 24;7(41):67150–65. doi: 10.18632/oncotarget.11567 (PMC5341864; doi:10.18632/oncotarget.11567)
Supplement: Supplementary file 1 [file oncotarget-07-67150-s001.pdf]

Angiotensin II type I receptor (AT1R) is an independent prognosticator of esophageal squamous cell carcinoma and promotes cells proliferation via mTOR activation

SUPPLEMENTARY FIGURES

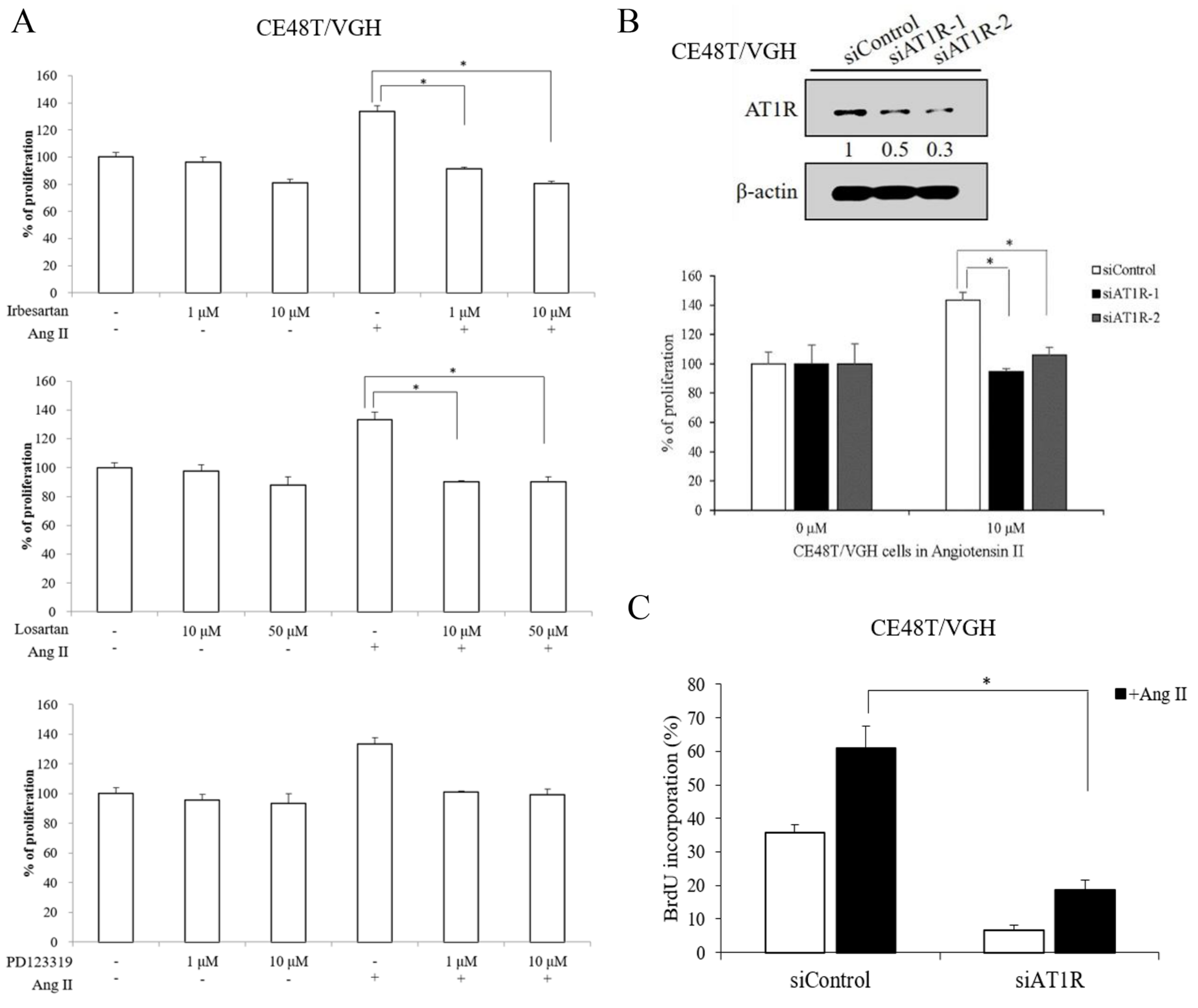

Supplementary Figure S1: AT1R expression involved in angiotensin II-induced cell growth in CE48T/VGH. A. Serum-starved CE48T/VGH cells were pre-treated with or without indicated concentrations of irbesartan or losartan or PD123319; the cells were then stimulated with angiotensin II. The MTT assay was performed to quantitate cell growth. **B** and **C.** The abilities of cell proliferation and BrdU incorporation in AT1R-knockdown CE48T/VGH cells or siControl group were assayed in the absence or presence of angiotensin II stimulation.

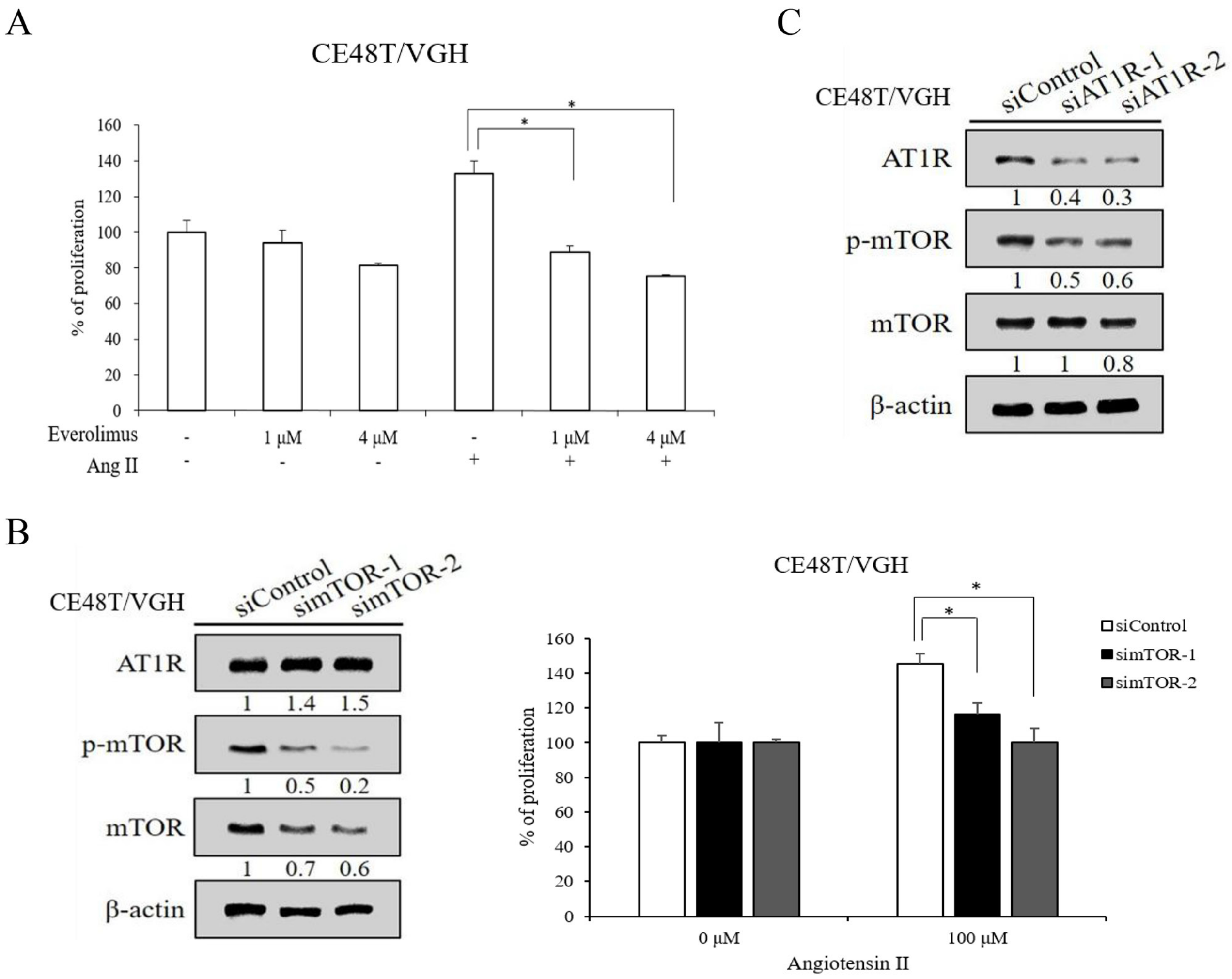

Supplementary Figure S2: The mTOR expression or activity was required for angiotensin II/AT1R signaling in CE48T/VGH cells. A. Serum-starved CE48T/VGH cells were pre-treated with indicated concentrations of everolimus followed with or without angiotensin II stimulation. The cell growth capabilities were determined by MTT assay. **B.** The protein expression levels of total mTOR, phosphorylated mTOR, and AT1R were demonstrated in CE48T/VGH cells transfected with siControl and simTOR. The cell growth capabilities of siControl and simTOR stimulated with angiotensin II were measured by MTT assay. **C.** The protein expression profiles of AT1R, total mTOR, and phosphorylated mTOR were determined in AT1R-depleted CE48T/VGH cells.
